# Variable manifestations, diverse seroreactivity and post-treatment persistence in non-human primates exposed to *Borrelia burgdorferi* by tick feeding

**DOI:** 10.1371/journal.pone.0189071

**Published:** 2017-12-13

**Authors:** Monica E. Embers, Nicole R. Hasenkampf, Mary B. Jacobs, Amanda C. Tardo, Lara A. Doyle-Meyers, Mario T. Philipp, Emir Hodzic

**Affiliations:** 1 Division of Bacteriology and Parasitology, Tulane National Primate Research Center, Tulane University Health Sciences Center, Covington, LA, United States of America; 2 Division of Veterinary Medicine, Tulane National Primate Research Center, Tulane University Health Sciences Center, Covington, LA, United States of America; 3 Center for Comparative Medicine, Schools of Medicine and Veterinary Medicine, University of California Davis, Davis, CA, United States of America; University of Kentucky College of Medicine, UNITED STATES

## Abstract

The efficacy and accepted regimen of antibiotic treatment for Lyme disease has been a point of significant contention among physicians and patients. While experimental studies in animals have offered evidence of post-treatment persistence of *Borrelia burgdorferi*, variations in methodology, detection methods and limitations of the models have led to some uncertainty with respect to translation of these results to human infection. With all stages of clinical Lyme disease having previously been described in nonhuman primates, this animal model was selected in order to most closely mimic human infection and response to treatment. Rhesus macaques were inoculated with *B*. *burgdorferi* by tick bite and a portion were treated with recommended doses of doxycycline for 28 days at four months post-inoculation. Signs of infection, clinical pathology, and antibody responses to a set of five antigens were monitored throughout the ~1.2 year study. Persistence of *B*. *burgdorferi* was evaluated using xenodiagnosis, bioassays in mice, multiple methods of molecular detection, immunostaining with polyclonal and monoclonal antibodies and an *in vivo* culture system. Our results demonstrate host-dependent signs of infection and variation in antibody responses. In addition, we observed evidence of persistent, intact, metabolically-active *B*. *burgdorferi* after antibiotic treatment of disseminated infection and showed that persistence may not be reflected by maintenance of specific antibody production by the host.

## Introduction

A growing public health problem, Lyme disease (LD) is the most commonly reported vector-borne disease in the U.S. and Europe [1], inflicting a significant public health burden [2–4]. The number of annual cases of LD in the U.S. has climbed to over 300,000 [5] and is expected to rise [6, 7]. Human infection with the etiologic agent of Lyme disease, *Borrelia burgdorferi*, results in disease of a few hallmark clinical signs and multifarious symptoms. The most common early manifestation of Lyme disease is an erythema migrans (EM) rash at the site of the tick bite. A large clinical study of disease characterization indicated that this occurs in 70–80% of cases [8]. However, variations in rash appearance [9], region [9, 10], and possibly even gender [11] may influence the accurate reporting of EM incidence. Following hematogenous dissemination of the spirochete, the heart, joints and nervous system may become colonized, leading to inflammation and signs/symptoms associated with this systemic infection.

The majority of LD cases that are readily diagnosed and treated lead to clinical cure. However, a proportion of patients remain ill [12, 13], and delayed treatment is associated with negative clinical outcomes [14, 15]. Through the use of animal models, researchers have sought to understand the etiology of post-treatment Lyme disease syndrome (PTLDS), namely whether or not the spirochetes persist post-treatment and could thus contribute to chronic symptoms. The recovery of attenuated, non-cultivable *B*. *burgdorferi* and the observation of remnants within joint tissue from antibiotic-treated mice have generated confusion as to the role of this persistent pathogen in PTLDS [16–19]. A landmark study published in 2014 [20] demonstrated the resurgence of *B*. *burgdorferi* growth in mice 12 months after antibiotic treatment. These findings lend credence to the viability of slow-growing spirochetes that persist after treatment.

Mouse models have provided key findings on pathogen infectivity and host responses to infection [21–25]. A variety of mouse strains and *B*. *burgdorferi* variants have been used for this purpose, whereby researchers can control the genetics of both the host and pathogen. In human infection, host genetics vary widely, multiple variants of *B*. *burgdorferi* may be the infecting pathogens, co-infections could contribute to disease presentation, and the possibility of re-infection cannot be restricted; thus, neither host nor pathogen can be controlled. Nonhuman primates (NHPs) are outbred and offer us the ability to control the infection with a single well-defined pathogen and examine responses that are due to host variability. In this study, we have been able to identify both similarities and differences in response to *B*. *burgdorferi* infection using this non-human primate model.

While mice, guinea pigs, dogs, rabbits and monkeys have been used as animal models to study *B*. *burgdorferi* infection, rhesus macaques have been shown to most closely recapitulate the multi-organ nature and progression of human LD [26, 27]. Characteristics of human disease such as erythema migrans, carditis, arthritis, and neuropathy of the peripheral and central nervous systems have all been observed in macaques [28]. In mice, the major reservoir host for *B*. *burgdorferi*, disease varies by mouse strain and age, while the early and late-disseminated manifestations are either absent or temporary [29]. Neurological disease from *B*. *burgdorferi* infection is also absent in rodents, rabbits and dogs [30]. Rhesus macaques exhibit the signs characteristic of the three phases of LD, including early-localized, early-disseminated, and late-stage Lyme disease [26, 31–34]. As in humans, spirochetes disseminate from the site of inoculation to multiple organs. Following experimental inoculation and progression, macaque tissues shown by PCR to harbor *B*. *burgdorferi* include skeletal muscles, heart, bladder, peripheral nerves, and the central nervous system (cerebrum, brainstem and cerebellum, spinal cord, and dura mater). Collectively, the NHP studies convincingly demonstrate that the macaque model most closely resembles human borreliosis and provides the best experimental model to examine post-treatment persistence and associated disease.

In a previous study, we infected macaques with *in vitro*-cultured *B*. *burgdorferi* using high dose inocula by syringe 4–6 months prior to treatment [35]. Persistent spirochetes were detected in some animals by xenodiagnosis, but their viability was uncertain. We hypothesized that inoculating animals by tick would produce the same result when treatment was delayed into the disseminated phase. We further hypothesized that the fitness of persistent *B*. *burgdorferi* obtained from treated and untreated animals would be evident as differences in infectivity when injected into mice. In this report, we describe the variations in serum antibody responses and disease manifestations in rhesus macaques infected with *B*. *burgdorferi* by tick bite. We also provide evidence for persistence of the pathogen post-treatment by multiple methods. While infection of mice with *B*. *burgdorferi* recovered from any monkey (treated or untreated) was not generally productive, the recovery by xenodiagnosis and through the use of an *in vivo* culture system, of intact spirochetes transcribing RNA indicates that the persistent spirochetes recovered post-treatment are of comparable viability to those recovered from untreated primates.

## Materials and methods

### Animals and inoculations

Practices in the housing and care of nonhuman primates, mice and rats conformed to the regulations and standards of the Public Health Service Policy on Humane Care and Use of Laboratory Animals, and the Guide for the Care and Use of Laboratory Animals. The Tulane National Primate Research Center (TNPRC) is fully accredited by the Association for the Assessment and Accreditation of Laboratory Animal Care-International. The Tulane University Institutional Animal Care and Use Committee approved all animal-related protocols, including the infection and sample collection from NHPs and the surgeries on rats. All animal procedures were overseen by veterinarians and their staff. Monkeys were pair-housed at all times, except when tick containment devices and jackets were in use; for that period, paired monkeys were in protected contact. Macaques received food (monkey chow) and water *ad libitum*, and standard enrichment (food supplements, manipulatable items in cage, human interaction with caretakers, perches or swings). Routine husbandry practices include the reporting of any abnormal clinical sign or activity by animals to the appropriate veterinary medical staff and faculty. Animal Care Technicians provided support during diagnostic and therapeutic procedures and the administration of the preventive medicine program. The Unit of Animal Resources provided assistance, equipment and support for the project work. The Unit provides after-hours care, which includes administration of treatments, collection of biologic samples for research activities and observation of animals. Any non-compliance with drug (antibiotic) treatments would have been reported to the assigned veterinarian, and this did not occur. The standard method of euthanasia at the TNPRC, and that which was used, is anesthesia with ketamine hydrochloride (10 mg/kg) followed by an overdose with sodium pentobarbital. This method is consistent with the recommendation of the American Veterinary Medical Association guidelines.

Mice were caged in groups of no more than five, fed mouse chow and given water *ad libitum*, and provided huts, bedding and chewable items for enrichment. SCID mice were kept in microisolator cages. Their food, water and cage bedding were autoclaved prior to use. All mouse manipulations were done under a laminar-flow hood. Rats were singly housed, fed rodent chow, given water *ad libitum*, and provided huts, bedding, chewable items, and cereal for enrichment. All surgeries were performed by a veterinarian. Mice and rats were euthanized with CO_2_ inhalation.

**Fig 1:**
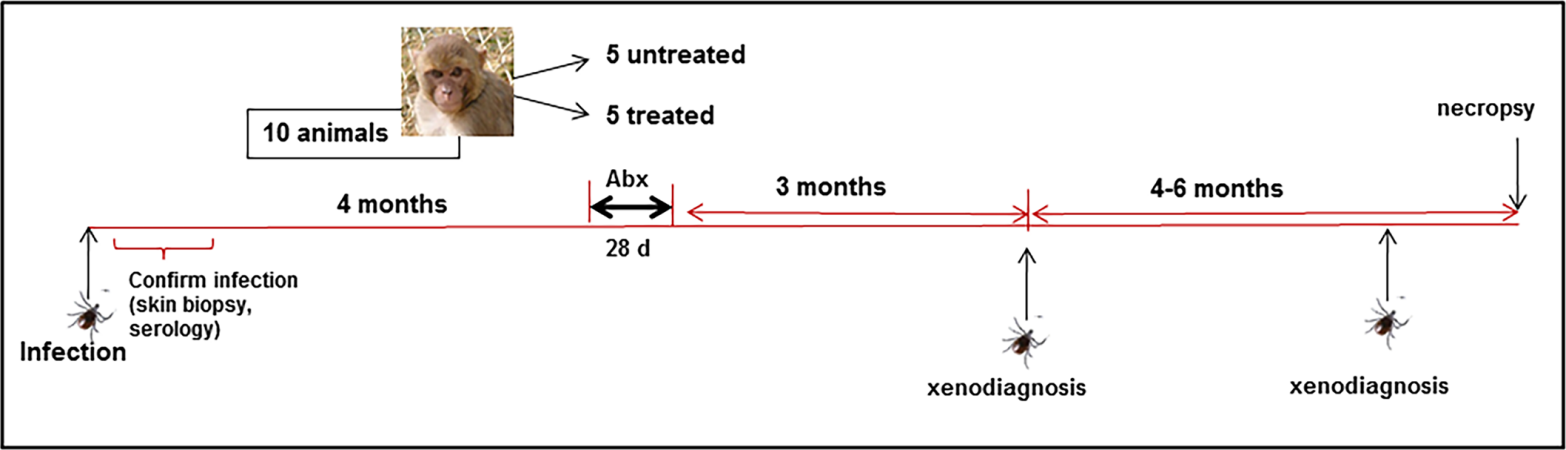
Experimental design for evaluation of antibiotic treatment following tick-mediated infection. Xenodiagnoses were conducted with uninfected nymphal ticks.

Ten male Indian rhesus macaques (*Macaca mulatta*), aged 2–3 years were subjected to infection with *B*. *burgdorferi* by nymphal *Ixodes scapularis* tick feeding. Treatment groups were randomly assigned prior to infection. A total of 20 ticks harboring *B*. *burgdorferi* strain B31.5A19 [36] were placed on each macaque. This technique, using ticks generated by the Vector-borne Diseases Core at the TNPRC, is described in detail elsewhere [37]. The ticks that fed on animals were crushed and inspected by direct fluorescence assay (DFA) for *B*. *burgdorferi*, described below. One hundred percent of the ticks were positive, due to the fact that they were individually capillary tube-fed the *B*. *burgdorferi* prior to placement on monkeys. If fewer than 50% of ticks (10) fed on each animal, more infected ticks were added to achieve a 50% feeding rate. At one and two weeks after the completion of tick feeding, 4 mm skin biopsies were taken for PCR and culture. The wounds were sealed with skin glue. Skin biopsies were assessed for infection by culture and by standard PCR using primers targeting *flaB*, *ospA* and *ospC* as described [35]. Blood was collected at multiple time points for serology (below). A synopsis of the experimental design is shown in Fig 1. Statistical analyses were conducted with GraphPad Prism software.

### Antibiotic treatment and monitoring

At 16 weeks post-tick feeding/inoculation, five of the ten monkeys received 25 mg doxycycline hyclate tablets, twice per day (6:30–8:30 pm and 7:30–9:30 am). This corresponded to 4.2–5.7 mg/kg [38], depending on the animal’s weight at the time of treatment. The human dose (100 mg) for a 150 lb. individual would be 1.47 mg/kg, but our pharmacokinetic study indicated that in macaques, a dose of 5 mg/kg is to achieve MIC levels in blood for over 80% of the treatment duration of 12 hours. In the 2 weeks prior, these animals were trained to accept tablets within treats. Blood was collected at day 10 and day 24 of the 28-day treatment regimen to determine serum antibiotic levels. These collections were taken in the morning (~9 am) before antibiotic treatment was given (as food is withheld prior to anesthesia), so thus represent 12+ hour trough levels. Serum doxycycline levels were measured by a modified Kirby-Bauer assay, as described [35]. Briefly, *B*. *subtilis* was used as the indicator strain with 75 μl serum per 6 mm disc and with doxycycline standards between 0.1–10 μg/ml. The detected antibiotic levels using this method are shown in S1 Table. With the exception of IK14 (0.1–0.26 μg/mL), the values obtained (0.98–1.87 μg/mL) were above or consistent with expected trough values derived from our previous pharmacokinetic study [38].

### Clinical laboratory tests and physical examination

At day 0 and every 2 months following tick inoculation, whole blood was collected for complete blood counts, including an erythrogram, leukogram, and differential cell count (percentage and absolute leukocyte count). Clinical chemistry evaluation included alanine aminotransferase (ALT), aspartate aminotransferase (AST), total protein, albumin, globulin, albumin/globulin (A/G) ratio, blood urea nitrogen (BUN), creatinine, BUN/creatinine ratio, glucose, sodium, potassium, and chloride.

#### Serology

Blood was collected for serum on day 0, every 2 weeks for 5 months, then monthly, in addition to the collections between weeks 16–20 for antibiotic-treated animals. Longitudinal antibody responses targeting five different *B*. *burgdorferi* antigens (OspA, OspC, DbpA, OppA-2 and the C6 peptide [39]) were measured by a recently-described Luminex^®^-based assay [40]. Samples from each time point were analyzed in triplicate.

#### Xenodiagnosis

Unfed ticks were placed on monkeys on day 240 post-inoculation (p.i.) and once again between days 320–360 (p.i.). These time points corresponded to ~3 months and 6–7 months after the completion of doxycycline treatment (8 and 12 months p.i., respectively). For each round of xenodiagnosis, 20 uninfected *Ixodes scapularis* nymphs were placed on each monkey. This procedure is described in a video journal format [37]. Between 7–13 ticks fed on each monkey. Between 10–14 days after drop-off, tick contents were processed individually for immunofluorescent staining and culture, and pooled for nucleic acid detection, as described [35]. Ticks were washed with 1% sodium hypochlorite, 0.5% benzalkonium chloride and 70% ethanol for 5 min each and crushed in 50 μL PBS with a microvial pestle. For the first round of xenodiagnosis, the contents were split into three 15-μL portions for: (1) DFA; (2) culture; and (3) RT-PCR. Following the second round of xenodiagnosis, a portion of the tick contents were also pooled for injection into severe combined immune-deficient (SCID) mice instead of PCR. Here, tick midguts were pooled per animal, pelleted, washed with 5 mL HBSS and then resuspended in HBSS in a final volume of 0.25 mL for mouse injections. For DFA, the samples were smeared on microscope slides, dried and fixed with acetone. The samples were stained with an anti-*Borrelia sp*.*-* FITC antibody (KPL) and examined by fluorescence microscopy to detect the presence of spirochetes [35]. In addition to staining the midgut contents with FITC-labeled polyclonal anti-*Borrelia* species antibody, we washed and re-stained this set and stained the second set of xenodiagnostic tick (XT) samples with an anti-OspA monoclonal antibody (CB10, obtained from J. Benach [41]), followed by anti-mouse IgG-Alexa 488 (Molecular Probes). As a control for the infection status of the ticks, 10 nymphs each from the same cohort of ticks were fed upon three uninfected C3H/HeN mice. These ticks were processed for immunofluorescent staining and nucleic acids were extracted for use as PCR/RT-PCR controls. The stained slides were coded and inspected for green *B*. *burgdorferi* by a skilled technician who was blinded to the identity of the specimens. While we could not use the XT slides with secondary antibody only as a negative control (due to the limited material), we did stain smears of ticks fed on clean mice using all antibodies (primary and secondary). These were uniformly negative for bright green structures that resembled spirochetes. For culture, samples were added to ∼4 mL BSK-H medium and incubated for 8–9 weeks at 34°C, in the presence of 5% CO_2_ and influx of N_2_ to produce a microaerophilic environment. None of the xenodiagnostic tick cultures grew spirochetes (irrespective of treatment), but nonmotile spirochetes were apparent. We therefore pelleted the cultures (5000 xg, 20 min), resuspended the pellets in a small amount of PBS and placed the preparations on slides for drying, fixing and staining.

### Injection of tick contents into severe-combined immune-deficient (SCID) mice

After the second round of xenodiagnosis, one Cb17.SCID mouse (Charles River Labs) per monkey was injected with pooled tick contents. After 28 days, mice were euthanized and tissues were collected for organ culture and RT-PCR. *OspA* and *flaB* genes were subjected to RT-PCR as described [35]. Xenodiagnosis was also performed on a subset of the SCID mice (5 total) at 21 days post-injection of tick contents. These XT contents were subjected to culture and indirect fluorescence assay (IFA) using the anti-OspA monoclonal as above.

### Post-mortem tissue analysis

Following euthanasia, blood, CSF, and multiple tissues were collected. Those fixed for histology, placed in tissue culture, and snap-frozen in OCT for cryosectioning included the heart, pericardium, bladder, knee and elbow joint tissues, skeletal muscle (bicep and quadriceps), dura mater, brain (cerebrum, cerebellum and brainstem), back skin, axillary and mesenteric lymph nodes, spleen, spinal cord and dorsal root ganglia, and peripheral nerves (sural, tibial, ulnar and median). Each of these tissues was also processed fresh for culture in standard (BSK-H) medium. Cultures were kept for 9 weeks in a 37°C CO_2_/N_2_ incubator.

#### Pathology

Animal skin, joints and lymph nodes were evaluated at necropsy, and with the exception of some mildly enlarged lymph nodes, no gross pathology was evident upon necropsy. A general histopathology report was provided by one veterinary pathologist, and a more extensive analysis was performed by another. Only the primary findings are reported here and comprehensive analysis will be communicated in a separate report.

#### Molecular detection

For each animal, sections of heart, skeletal muscle, and lung were collected in RNAlater (Qiagen), stored at -20°C and later processed for RNA. DNA was also extracted from tissue samples of heart, skin, and those indicated as possessing inflammation in the histopathology report. *B*. *burgdorferi* DNA in these tissues was detected by a nested PCR program utilizing 5S and 23S-targeted primers [42]. This included external forward and reverse primers (CTGCGAGTTCGCGGGAGA-3’fwd; 5’-TCCTAGGCATTCACCATA-3’rev) and internal forward and reverse primers (5’-GAGTAGGTTATTGCCAGGGTTTTATT-3’fwd; 5’-TATTTTTATCTTCCATCTCTATTTTGCC-3’rev) targeting the 5S-23S intergenic sequence. Standard PCR and RT-PCR for *ospA*, *ospC* and *flaB* were also performed with either SCID mouse tissues or XT midguts using primers described elsewhere [35].

For relative quantitative analysis of mRNA levels of *B*. *burgdorferi* target genes, samples of the heart, meninges, skeletal muscle, axillary lymph node and lung were collected from each animal. The tissue samples were immediately weighed, snap-frozen in liquid nitrogen, and stored at −80°C before nucleic acid extraction. Total RNA was purified with RNeasy mini kits according to the manufacturer's instructions (Qiagen, Valencia, CA) using ~10 mg of tissue. Samples were first homogenized with a Geno/Grinder and then treated with RNase-free DNase I prior to elution. The concentration and purity of extracted RNA were determined by measuring the A260 and A280. The extracted total RNA was stored at −80°C until use. The extracted total RNA was subjected to two separate reactions—one to synthesize cDNA and the other to test for DNA contamination.

First-strand cDNA was synthesized from total RNA (~200 ng/μl) using the QuantiTect Reverse Transcription Kit (Qiagen, Valencia, CA) in 50-μL reactions, according to the manufacturer's instructions. The Advantage® 2 Polymerase Mix (Clontech Laboratories) was used to increase fidelity, efficiency, and greater yield of cDNA, according to the manufacturer's instructions, because of the limited numbers of spirochetes and spirochetal RNA (cDNA) in tissue samples. The pre-amplification reaction involved activation at 95°C for 15 sec, amplification for 25 cycles at 95°C for 15 sec, 55°C for 15 sec, and 70°C for 45 sec, followed by elongation at 70°C for 5 min. The pre-amplified products were diluted at a ratio of 1:10 and used as templates for qPCR analysis. All samples were analyzed for the presence of 18S rRNA to determine the efficiency of the nucleic acid extraction, amplification, and as an indicator of inhibition. For every run and for every gene of interest, several controls were utilized, including: 1) positive control *B*. *burgdorferi* RNA; 2) negative controls (a. no template control, b. unrelated template control); and 3) no RT control to check for DNA contamination. The specificity of each assay was determined using cDNA templates in duplicate from uninfected mouse tissues and other pathogens (*Leptospira spp*., *Escherichia coli*, *Listeria monocytogenes*, *Staphylococcus aureus*, *Helicobacter spp*.).

Three oligonucleotides, two primers, and an internal fluorescence-labeled probe for *B*. *burgdorferi* (strain N40) *ospA* and *oppA-2* target genes were designed with Primer Express software (Thermo Fisher Scientific). The amplification efficiency (E) of each assay was calculated from the slope of a standard curve generated on a 10-fold dilution in triplicate, using the formula E = 10(-1/slope) -1. In order to obtain accurate and reproducible results, all assays were determined to have an efficiency of >95%. The sensitivity and reproducibility of real-time PCR for each target gene were determined by spiking normal tissue samples with known numbers of spirochetes to establish standard curves. Each standard assay was performed in triplicate, with nearly identical results derived from replicate samples. The analytical sensitivity for each target gene RNA was in the range of 1 to 109 spirochetes, with a yield of detection close to 90% of the calculated amount of known target in each sample. All assays were performed with positive and negative control samples, and control results verified the validity of positive and negative findings. False-positive amplification was not detected (Cq values were always 40).

#### Immunofluorescent staining

Frozen tissue samples were sectioned at 16μm with a microtome cryostat (Cryostar HM 560 MV; Microm International GmbH) and placed on slides that were stored at -20°C. Tissue section samples were thawed and permeabilized with PBS containing 0.2% fish skin gelatin, 0.1% Triton X-100 (PBS/FSG/TX) containing 0.02% sodium azide (AZ) with gentle agitation for 1 hour. The TX-100 was washed out using PBS/FSG/AZ. Slides were kept in a dark humidified staining box for blocking and staining steps. After blocking for 1 hour with 10% normal goat serum (NGS), the slides were incubated with 200 μl primary antibody, either a polyclonal rabbit anti-Borrelia (Accurate chemicals, Westbury, NY) at 1:200 or the anti-OspA monoclonal, CB10, with a 1:30 dilution of hybridoma supernatant for 1 hour at room temperature (RT). After washing with PBS/TX using a disposable transfer pipet, slides were left in PBS/TX for 5 minutes at RT. Slides were rinsed with PBS/FSG and incubated for 45 minutes at RT with the secondary antibody (anti-mouse IgG-Alexa-488) diluted 1:1000 in PBS/NGS. The wash step with PBS/TX was performed and slides were mounted with anti-quenching reagent (Sigma Aldrich). Negative controls including the secondary antibody alone and uninfected monkey tissue were used, as was an *ex vivo* section of infected macaque brain tissue [43] for the positive control.

#### *In vivo* culture

Given the absence of *B*. *burgdorferi* growth with infected, immunocompetent macaque tissue specimens in standard BSK (*in vitro*) culture [35, 44], we devised an *in vivo* culture system. At necropsy, heart tissue was collected and placed on ice. An internal section of ~10 mm^3^ was cut with a sterile scalpel and placed into 5 ml of BSK-H (Sigma Aldrich) media within 8 kD MWCO dialysis tubing (Millipore). One section of heart from each monkey was used and each individual dialysis bag was implanted into the peritoneal cavity of a 7–8 week-old CD IGS rat (Charles River Labs). This was performed as described [45], but with some modifications. Rats were anaesthetized with isoflurane gas in an induction chamber first and then via nose cone through the entire procedure. Each animal was given an injection of local anesthetic (0.25% marcaine, 0.1cc) at the incision site. An area of the middle/lower abdomen was shaved and treated with appropriate skin disinfectant (Betadine®). Using strict aseptic technique, a small (~1.5–2 cm) incision was made in the abdomen of the animal using a sterile scalpel and forceps. The bag containing heart specimen and BSK-H medium was inserted and implanted in the peritoneal cavity. The incision was sealed by double layer suturing whereby the muscle layer was closed with polydiaxanone (PDS) and the skin layer with nylon, utilizing the smallest adequate gauge suture. Subcuticular sutures closed with PDS and skin glue was also used. As a pre-emptive analgesia, rats were given buprenorphine (0.05 mg/kg) via subcutaneous injection. After 20 days, rats were euthanized and the dialysis bags were recovered. Tissues were subjected to *in vitro* culture, RT-PCR and frozen for tissue sectioning and IFA. Pellets from the *in vitro* culture of heart tissue grown in dialysis membrane chambers (DMCs) were stained with either: (1) a combination of anti-OspA and anti-OspC [46] monoclonal antibodies, and after washing, the antibodies were detected with anti-mouse IgG2a-Alexa-fluor568 (red); or (2) anti-OspA alone, followed by anti-mouse IgG-Alexa fluor488 (green). Data are only available for 2 rats, as the contents of 2 DMCs were too contaminated to process.

## Results

### Productive infection in monkeys lacks objective signs and may be self-limited

**Fig 2:**
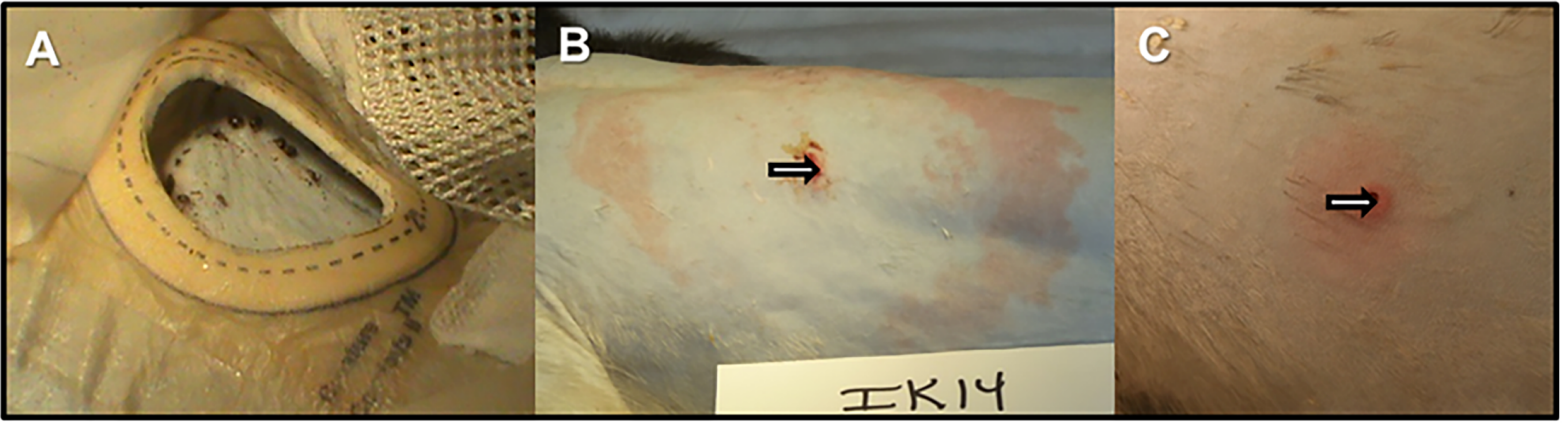
Tick-mediated inoculation of *B*. *burgdorferi* produces different levels of erythema in monkeys. Ticks were allowed to feed to repletion on the backs of rhesus macaques for infection (Panel A). One monkey produced a bona fide erythema migrans rash, seen one week after tick removal (Panel B), whereas the other monkeys produced only small, diffuse erythema at the site of tick feeding (Panel C).

After the animals had been fed upon by ticks, weekly observations and physical exams indicated mild to moderate axillary lymphadenopathy (S2 Table). In some cases, peripheral/inguinal lymphadenopathy was observed. Only one (IK14) of the 10 monkeys developed a *bona fide* erythema migrans lesion, while others showed diffuse erythema (Fig 2). Erythematous rashes distal from the tick feeding areas were observed in three monkeys at various time points post-infection. Skin biopsies from the sites of prior tick feeding were taken at 1 and 2 weeks following tick drop off and were subjected to culture and PCR (Table 1). Half of the animals produced positive skin biopsy cultures and 8/10 were PCR positive. Only one animal (IK14) was not positive by either test. CBC and serum chemistry was performed pre-infection and at either 2 or 4 months p.i. Only very moderate changes in liver transaminase levels and lymphocyte percentages in peripheral blood were observed ([47] and S2 Table).

**Table 1:** Culture and PCR results.

Animal #	Skin Biopsy Culture*	Skin Biopsy PCR*
**IK14**	**-**	**-**
**IL09**	**-**	**+**
**IH11**	**+**	**+**
**IK66**	**+**	**+**
**IH22**	**-**	**+**
**IL75**	**-**	**+**
**IP67**	**+**	**+**
**IN16**	**+**	**-**
**IN05**	**-**	**+**
**IP55**	**+**	**+**

*either 1 or 2 week time points

### Serological responses vary per detection antigen and with antibiotic treatment

**Fig 3:**
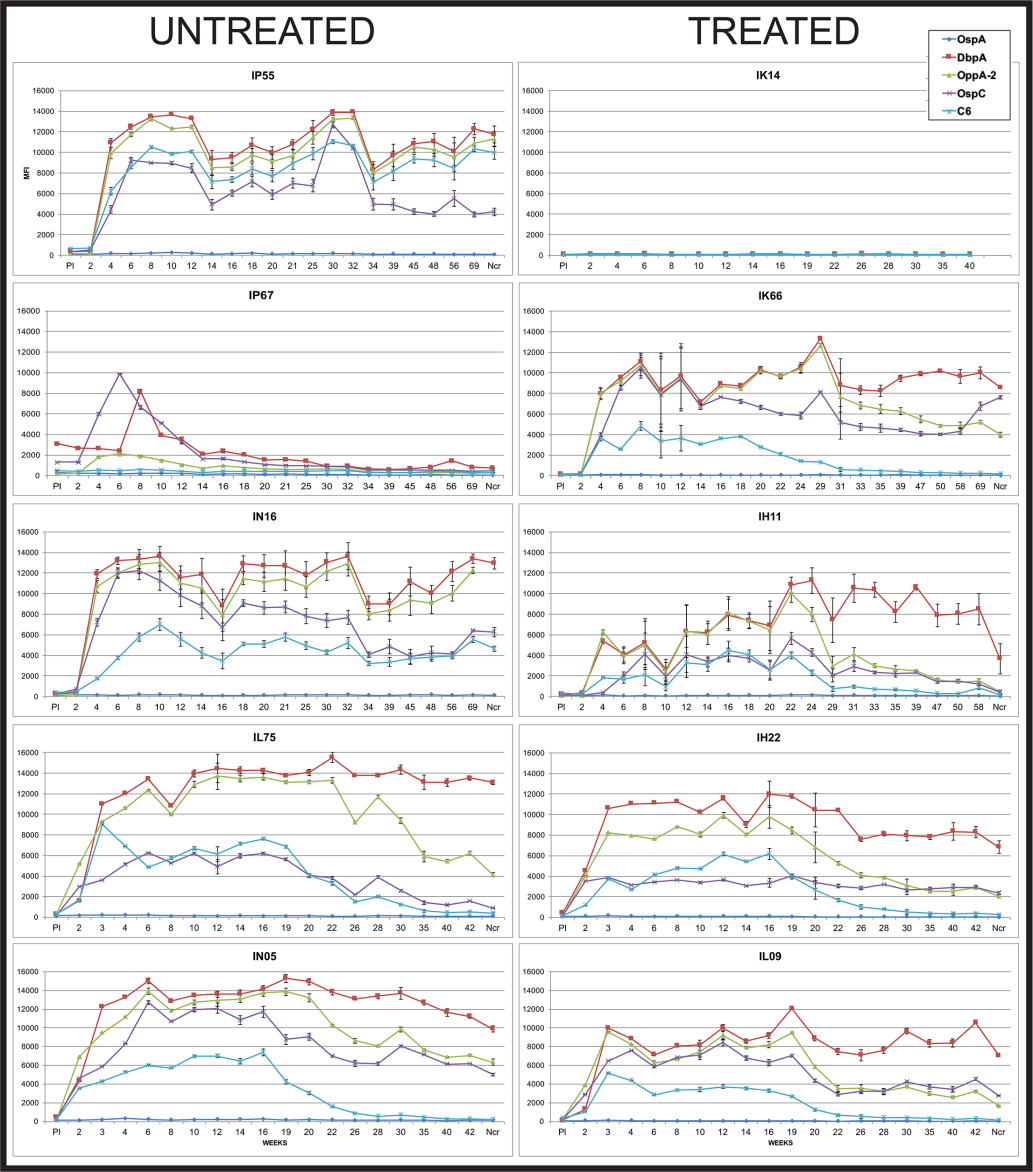
Longitudinal antibody responses to five *B*. *burgdorferi* antigens over the course of infection. Each graph represents one animal, with the untreated monkeys shown in the left column and the treated animals on the right. Animals were treated with doxycycline between weeks 16–20. Vertical axis: MFI = mean florescent intensity. Ncr = blood collected at necropsy. Shown is the mean ±SEM for each time point.

Blood was drawn for serum before, and throughout the course of infection. The responses to five *B*. *burgdorferi* antigens were measured using a Luminex^®^-based assay developed by our laboratory [40]. As expected, the C6 antibody levels waned after antibiotic treatment in those animals that received doxycycline at 16 weeks post-inoculation (Fig 3). A decline in C6 levels was also observed in 2 of the 4 untreated monkeys that developed anti-C6 responses, as observed previously [35]. In the untreated group, one monkey (IP67) developed a response to 3 of the 5 antigens early, but by weeks 14–20, the responses had declined to near pre-immune levels. This particular animal possessed antibodies to DbpA, OspC and OspA at the pre-immune time point. Given that our animals are housed outdoors prior to study assignment, the possibility of pre-exposure to a *Borrelia* species cannot be excluded. Cross-reactivity with intestinal spirochetes is a possible explanation as well. The remaining four untreated monkeys generated responses to OspC, OppA-2, C6 and DbpA; the antibody levels to DbpA and OppA-2 remained high throughout the infection period [48]. One of the treated monkeys (IK14) remained seronegative to all antigens throughout the study period. Interestingly, this was also the animal that developed an EM lesion. Western blots for IgM and IgG antibody detection indicated seronegativity against *B*. *burgdorferi* whole cell lysate (S1 Fig). With the exception of C6 responses, the remaining four treated monkeys demonstrated variability with respect to declines in antibody levels. For example, animal IH11 exhibited a drop to near pre-immune levels in the antibodies targeting OspC, OppA-2 and even DbpA. Conversely, monkey IK66’s responses to those antigens remained elevated throughout the study period (>1.2 years). Antibody responses to OspA were uniformly weak, likely due to the use of tick-mediated inoculation. We did not observe increases in anti-OspA levels later in infection or post-treatment (S2 Fig).

### Detection of *B*. *burgdorferi* in xenodiagnostic ticks indicates persistence of the spirochetes in treated and untreated monkeys

**Fig 4:**
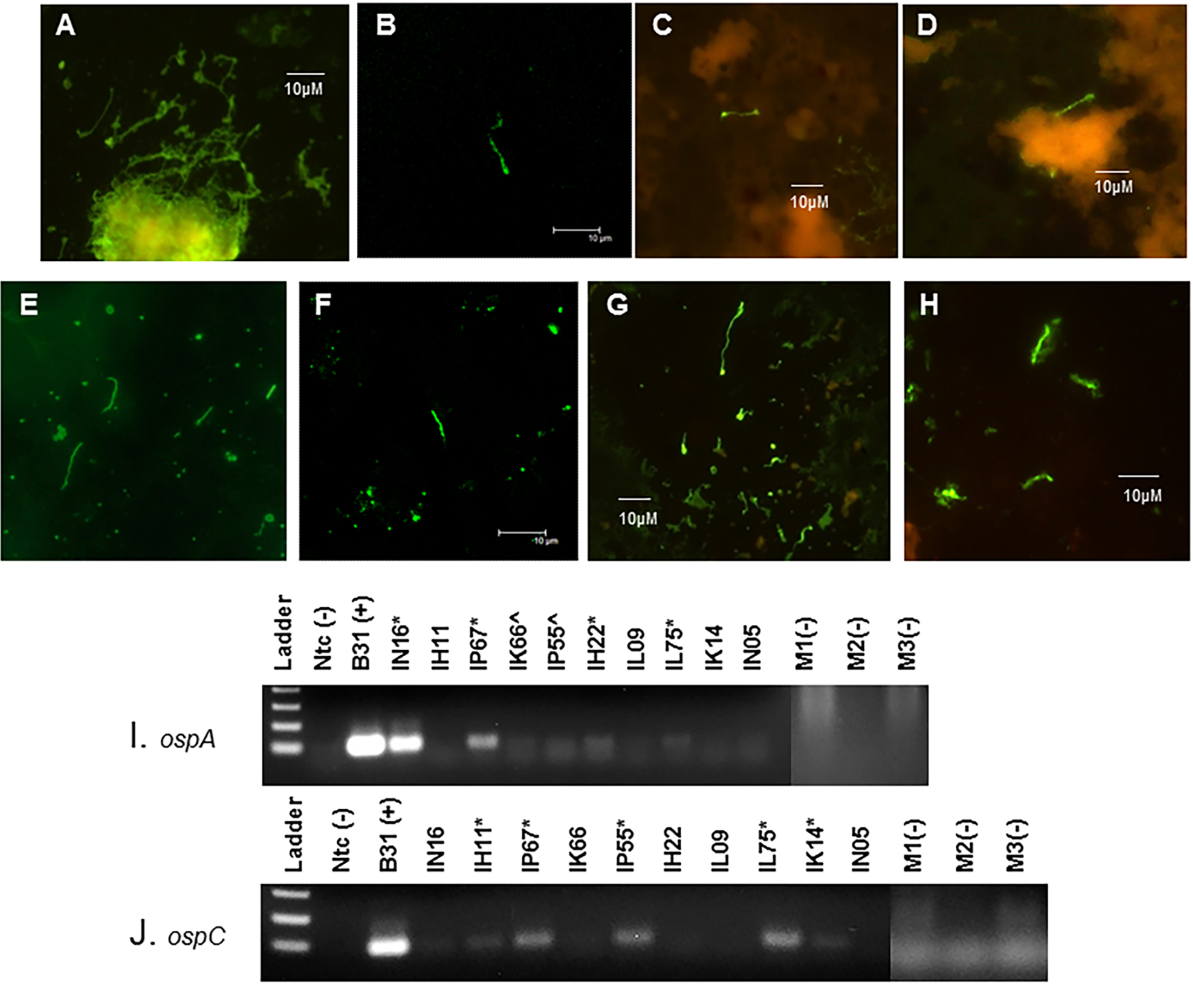
Detection of *B*. *burgdorferi* within XT by immunostaining and RT-PCR. Immunostaining reveals *Borreliae* within XT from both treated and untreated monkeys. Tick midguts were stained for IFA with an anti-OspA monoclonal antibody, followed by an Alexa-IgG-488(green)-conjugated secondary antibody (B,C and D) or stained for DFA using an anti-*Borrelia* sp. FITC-conjugated polyclonal antibody (E, F). In addition, cultures of tick midgut contents were pelleted and stained for IFA (G, H). Panel A = positive control for IFA using *B*. *burgdorferi* culture; Panel B = XT from animal IH11 (treated); Panel C = XT from animal IK14 (treated); Panel D = XT from animal IL09 (treated); Panel E = positive control for DFA using midgut smear of tick that was capillary tube-fed *B*. *burgdorferi*; Panel F = XT from animal IP55 (untreated); Panel G = XT culture pellet from animal IP55 (untreated); Panel H = XT culture pellet from animal IK14 (treated). RT-PCR for *ospA* (I) and *ospC* (J) transcripts indicate the presence of viable *B*. *burgdorferi*. RNA was extracted from XT following the first xenodiagnosis (~7 months p.i.). Cohort-matched control XT derived from feeding on clean mice were used and are indicated by m1, m2 and m3.). Clear positives are marked with (*) and potential positive results are marked with (^).

Given that we inoculated the monkeys by tick bite, the xenodiagnostic tick (XT) feeding represented a second exposure to ticks. This did induce erythematous papules at the site of the tick bite (S3 Fig), showing some anti-tick immunity. We performed xenodiagnosis twice, at ~3 and 7–8 months post-treatment. A pilot study conducted by S. Narasimhan (Yale University) and M. Philipp showed that multiple tick feedings did not affect transmission in rhesus macaques, but the impact of this on xenodiagnosis was uncertain. Initially, we stained the XT midgut smear using DFA with polyclonal anti-*Borrelia* species antibodies. As such, we found few positives. After the second xenodiagnosis, we stained the slides using IFA with a monoclonal anti-OspA antibody and found ticks from multiple monkeys to stain positive for *B*. *burgdorferi*. A portion of the XT midgut contents were also placed in culture. After several weeks, non-motile bodies with spirochete-like morphology were observed. We thus concentrated them by pelleting the culture and resuspending the pellet in a low volume; these samples were then stained for IFA. The results are shown in Fig 4. The tick midgut contents were also subjected to RT-PCR. The presence of *ospC* and *ospA* transcripts within XT that fed upon untreated monkeys IN16, IP67, IP55 and IL75 was clear, indicating that viable spirochetes were obtained. Less clear, but probable bands were also seen for a few of the ticks fed upon treated monkeys IH11, IK14 and IH22 (Fig 4 and Table 2).

**Table 2:** Summary of results.

Animal #	Treatment	Serology (longitudinal)	Xenodiagnosis			*In vivo* culture		PCR/RT-PCR	
		OspA(A) OspC (C) OppA2 (O) DbpA (D) C6	7 mo.	12 mo.	XT RT-PCR	IFA	Nested RT-PCR	5s_23s PCR	Nested RT-PCR
**IK14**	Doxycycline 28 days	(-)		(+) P,T	(+) *ospC*	(-)	(-)	(-)	(-)
**IL09**	Doxycycline28 days	(+)C,O, D,C6 ↓in C6		(+) T	(-)	(+)	(-)	(+)	(-)
**IH11**	Doxycycline 28 days	(+)C,O, D,C6 ↓in C6; only D+ at necr.	(+) T		(+) *ospC*	(+)	$\bar{X}C_{q}$ = 16.50 (*ospA*)	(+)	(-)
**IK66**	Doxycycline 28 days	(+)C,O, D,C6 ↓in C6, ↑in C	(+) T		(+) *ospA*	(+)	$\bar{X}C_{q}$ = 21.0 (*ospA*)	(-)	(-)
**IH22**	Doxycycline28 days	(+)C,O, D,C6 ↓in C6		(+) P	(+) *ospA*	(-)	(-)	(-)	(-)
**IL75**	No treatment	(+)C,O, D,C6 ↓in C6 and C		(+) T	(+) *ospA*, (+) *ospC*	(-)	(-)	(-)	(-)
**IP67**	No treatment	(+)C, D ↓ all by wk20	(+) T		(+) *ospA*, (+)*ospC*	(-)	(-)	(-)	(-)
**IN16**	No treatment	(+)C,O, D,C6 maintained	(+) T	(+) P	(+) *ospA*, (e) *ospC*	Contam.	(-)	(+)	(-)
**IN05**	No treatment	(+)C,O, D,C6 ↓in C6	(+) T	(+) T	(-)	(+)	$\bar{X}C_{q}$ = 22.88 (*oppA-2*)	(+)	(-)
**IP55**	No treatment	(+)C,O, D,C6 maintained	(+) T	(+) P,T	(+) *ospA*, (+) *ospC*	Contam.	(-)	(+)	(-)

T = tick contents

P = pellet of tick content culture

$\bar{X}C_{q}$ = mean value for Q-PCR quantification cycle

### *B*. *burgdorferi* recovered from infected monkeys by xenodiagnosis do not appear to productively infect immunodeficient mice

Following xenodiagnosis, a portion of the tick contents were pooled and injected into immune-deficient CB17.SCID mice. After 3 weeks, the mice were euthanized and their tissues were subjected to culture, PCR and RT-PCR. In another experiment, tick contents from the second xenodiagnosis of 5 monkeys were inoculated into SCID mice and those mice were also subjected to xenodiagnosis. The contents of xenodiagnostic ticks fed upon SCID mice were subjected to culture and PCR/RT-PCR as well. For both experiments, tissues (ear skin, heart, bladder, spleen and tibiotarsal joints) of all mice injected with XT contents were uniformly culture-negative. In the first xenodiagnosis experiment, RT-PCR was performed using *ospA* and *flaB* primers. No samples were positive by *ospA* RT-PCR and only one sample (joint tissue from mouse injected with XT contents of monkey IN16) was positive by *flaB* RT-PCR (S4 Fig). The monkey from which these XT were derived was untreated. In the second experiment, RNA from several mouse tissues was amplified with *ospA* primers. The XT from SCID mice injected with monkey XT contents were also uniformly negative by culture. Inspection by DFA did not reveal any bright green structures that resembled spirochetes. A number of green round bodies were observed, but without specific detection by molecular methods, we cannot assert that these are morphological variants of *B*. *burgdorferi*.

### Pathology is moderate and is found in multiple sites

**Fig 5:**
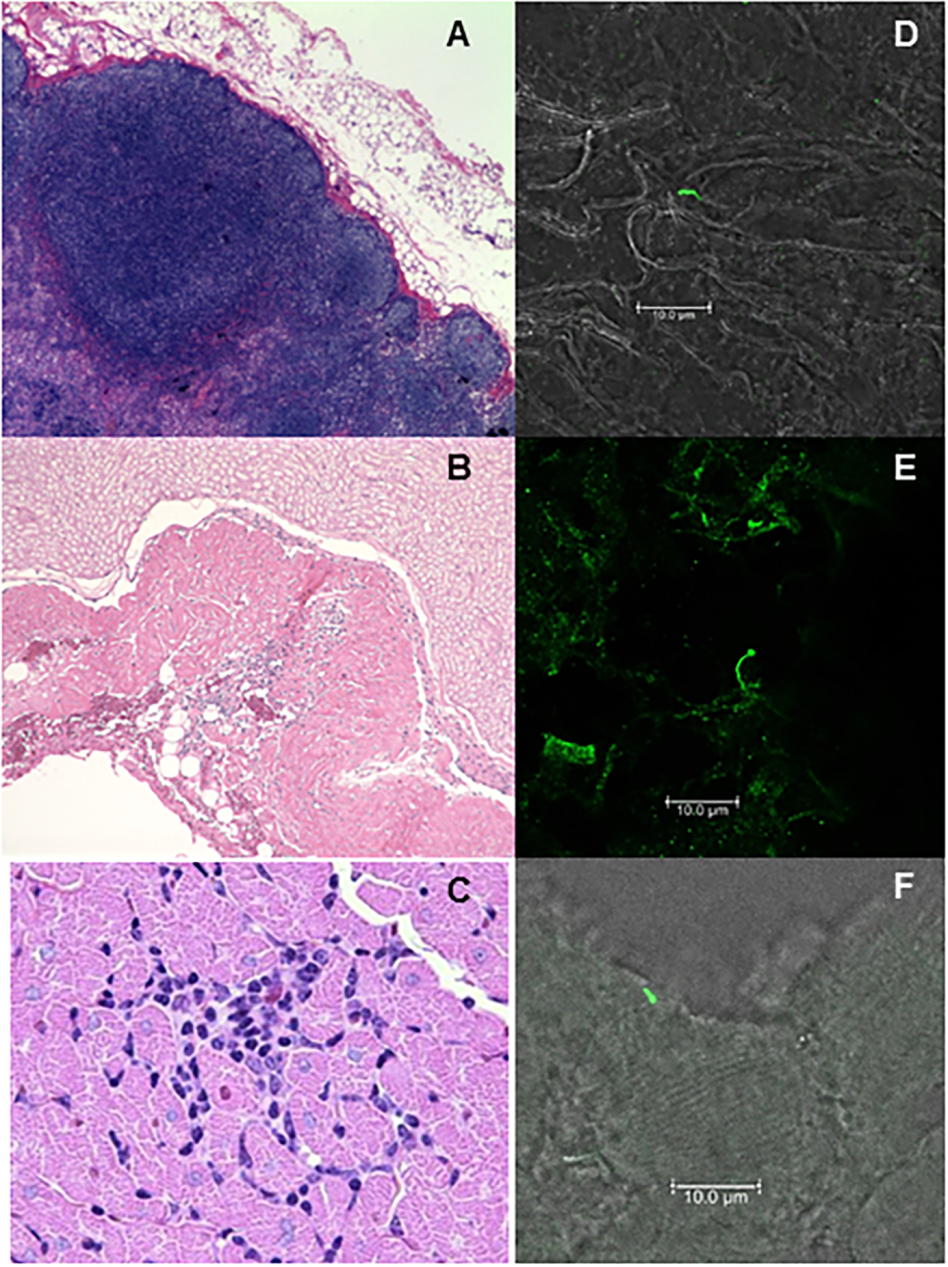
Histopathology and *B*. *burgdorferi* (Bb) within tissues of persistently-infected macaques. At 12–13 months post-infection, necropsy tissues were analyzed for evidence of tissue pathology. Frozen sections of affected tissues were then stained with fluorescently-labeled Borrelia-specific polyclonal antibodies. Shown here is: axillary lymph node hyperplasia and histiocytosis-200X (A) and Bb antigen (D) in untreated animal IN16; cervical spinal cord, focal inflammatory lesion-200X (B) and Bb antigen (E) from treated animal IH11; and a focal area of mononuclear inflammation in the myocardial interstitium-1000X (C) and Bb antigen (F) from untreated animal IN05.

Here we report on the general features of histopathology, including small pockets of inflammation and detection of *B*. *burgdorferi* with specific antibodies. Extensive evaluation of pathology and detection of *B*. *burgdorferi* within tissues is too sizeable to include in this report, so will be communicated separately. In brief, the affected tissues varied between animals, but inflammation was observed surrounding spinal cord, in the heart, skeletal muscle, and joints. Hyperplasia was observed in the lymph nodes and spleens of several monkeys as well. Small foci of inflammation in the lung were observed in some animals, but this did not appear to be specific, when comparisons to uninfected monkeys were made. A summary of the initial pathology report is provided in S2 Table, and a sample of images from this report is included in Fig 5. The inflammation was found to be minimal to moderate and focal in nature.

### Spirochetes are rare in affected tissues

Fresh frozen sections of affected tissues were also obtained for indirect immunofluorescent (IFA) staining. Ten sections were made and every third section was stained for IFA. In approximately 100 sections, 7 spirochetes were identified and this included tissues from both treated and untreated animals. Representative images are shown in Fig 5.

### Molecular detection of *B*. *burgdorferi* in necropsy tissues was infrequent or absent

**Fig 6:**
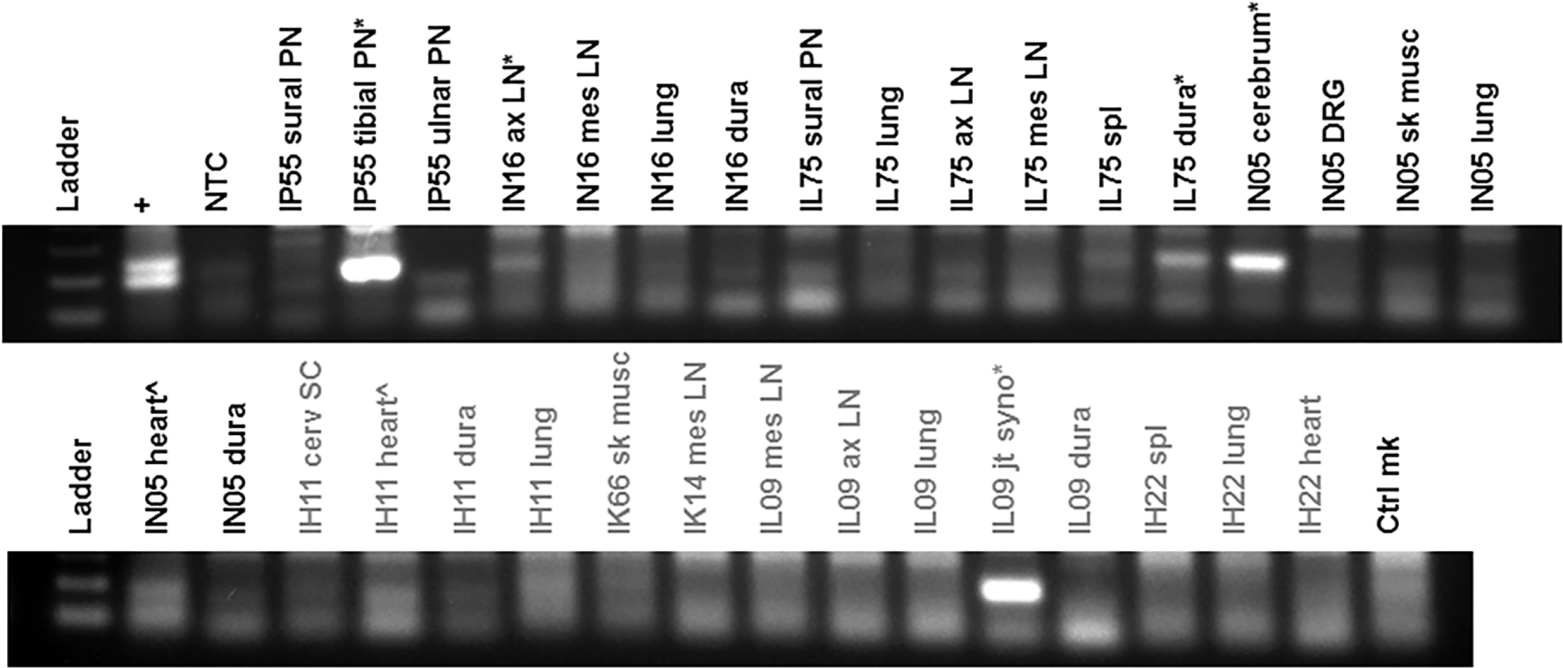
Amplification of *B*. *burgdorferi*-specific DNA in tissues showing histopathology. Tissue DNA was subjected to amplification of a region within the 5s-23s ribosomal genes using a nested set of primers. The positive control (+) consisted of B31.5A19 DNA and negative controls were no template control (NTC) and uninfected monkey skin tissue DNA (Ctrl mk). Tissues tested included peripheral nerves (PN), axillary (ax) and mesenteric (mes) lymph nodes (LN), lung, meninges/dura mater (dura), spleen, cerebrum, skeletal muscle (sk musc), heart, spinal cord (SC), and joint synovium (jt syno). Clear positives are marked with (*) and potential positive results are marked with (^). The treated monkeys are labeled with grey text.

Only tissues that were listed in the primary pathology report as having an anomaly (inflammation or hypercellularity) were subjected to molecular detection. The tissues were tested by a nested quantitative RT-PCR targeting the *ospA* and *oppA-2* genes [18] and also by standard PCR targeting a specific region of *B*. *burgdorferi* ribosomal gene sequences. The qRT-PCR targets of *ospA* and *oppA-2* were selected because transcripts of these genes have each been previously detected in antibiotic treated animals. In mice, *oppA-2* transcript [18] and in monkeys, *ospA* transcripts [35] were evident, where *flaB* was undetectable. We did not detect *B*. *burgdorferi* in frozen tissues using RT-PCR. This technique utilized 250 ng of RNA for the first step, which may have been too little given the size of monkeys and volume of tissue. For standard PCR, 500 ng of DNA from each tissue was added to the amplification. As shown in Fig 6, this produced clear positive and weak positive results. *B*. *burgdorferi*-specific DNA was amplified from tissue of untreated (IP55, IN16, IL75 and IN05) and treated (IH11 and IL09) animals.

### *In vivo* culture indicates persistence of metabolically active *B*. *burgdorferi* spirochetes in the heart tissue of treated and untreated monkeys

**Fig 7:**
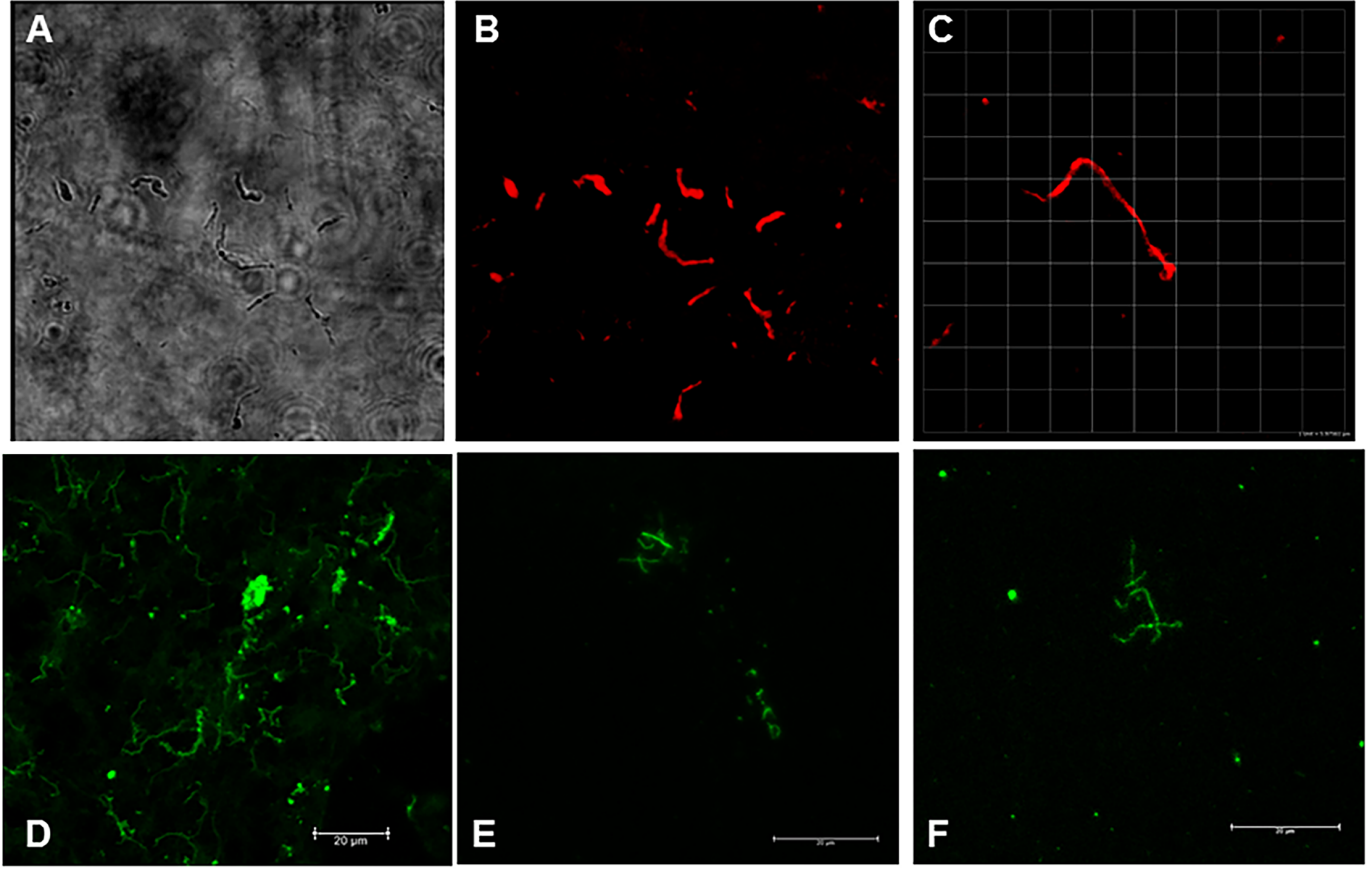
Spirochetes identified from *in vivo* culture of monkey heart tissue. The DMC-cultured heart tissue was placed in BSK and back to *in vitro* culture for 1 week. The culture pellets were then stained with a combination of anti-OspA and anti-OspC monoclonal antibodies, followed by a red detection antibody (Panels A,B,C) or with anti-OspA followed by a green detection antibody (Panels D,E,F). Shown are differential interference contrast (Panel A) and fluorescent detection (Panels B and C) of cultures stained with monoclonal antibodies. Panels A and B: animal IN05 (untreated); Panel C: animal IH22 (treated). Panel D is a positive control *B*. *burgdorferi* culture; Panels E and F are stained specimens from heart cultures of monkeys IK14 and IL09 (both treated), respectively.

As expected, culture of tissues from infected monkeys (treated or untreated) did not yield motile spirochetes. We surmised that the long period of host adaptation could result in a phenotype that prevents propagation in a nutrient-rich microaerophilic environment. We thus aimed to grow any persistent *B*. *burgdorferi* present in the heart tissue of our infected macaques within an isolated system exposed to a mammalian host environment. Heart tissue was placed in DMCs and implanted into the peritoneal cavities of rats for a period of 20 days. In some cases, the tissue disintegrated, and in some cases, it remained intact. However, by this point the tissue became necrotic. The media from the tissues was pelleted and stained for detection of *B*. *burgdorferi*. As shown in Fig 7, spirochetes were identified from this *in vivo* culture system. Eight of ten were successfully cultured and tissue was also sent to UC Davis for (blind) processing of RNA and detection of *B*. *burgdorferi*-specific transcription. From those eight samples, three were found to transcribe either *ospA* or *oppA-2* (Table 2). These included animals IN05 (untreated), IH11 (treated), and IK66 (treated). *B*. *burgdorferi* was detected by IFA in 4 of the 8 samples, including all three which were RT-PCR positive.

In summary, no significant differences were identified between treated and untreated animals when the following were tested (by Chi squared analysis and Fisher’s exact test): (1) Number of animals positive for *B*. *burgdorferi* by PCR; (2) number of animals positive for *B*. *burgdorferi* by xenodiagnosis; and (3) number of animals positive for *B*. *burgdorferi* by RT-PCR, either directly or by analysis of *in vivo* culture tissue. With respect to serology, a 4-fold drop in C6 titer by 30 weeks post-infection, comparing the untreated monkeys (2/5) and treated monkeys who seroconverted (4/4), was not significant (Fisher’s exact, p = 0.1667).

## Discussion

Upon inoculation of ten monkeys with *B*. *burgdorferi* by tick, only one developed a *bona fide* EM rash. What dictates the development of the characteristic rash in humans is not well understood, but could be affected by the genotypic variants of *B*. *burgdorferi* present in the tick, prior exposure, and possible co-infections. Notwithstanding, the frequency of this manifestation in macaques appears to be lower. Two differences in the comparison of experimental monkeys with human patients include the use of multiple ticks for infection and a lab-propagated strain of *B*. *burgdorferi*. We therefore do not place excessive significance on this finding. What is interesting about the monkey that developed EM is that his antibody response to *B*. *burgdorferi* was somehow absent, though we have evidence for persistent spirochetes by xenodiagnosis and presence in tissue (manuscript in progress). Perhaps the spirochetes remained localized in the skin for a longer period than the other monkeys and the adoption of an immunoevasive phenotype preceded dissemination. Regrettably, the skin biopsies from this animal (IK14) were collected in the center of the EM and not the leading edge, which may have led to the absence of *B*. *burgdorferi* detection in these samples.

Some clinical studies of human patients have indicated that lymphopenia and elevated liver enzyme values are associated with Lyme disease [49, 50]. Our analysis of CBC and blood chemistry did not reveal any significant differences in monkeys before and after infection. Unfortunately, we did not measure C-reactive protein and serum amyloid in these monkeys. Earlier time points may have offered an indication of liver function in the acute phase [50], but persistent hepatic effects were unapparent by these tests.

As described in other reports [19, 51], we were able to identify *B*. *burgdorferi*-specific DNA in a variety of tissues from infected and infected/treated monkeys. Our inability to detect *B*. *burgdorferi* transcripts directly from necropsy tissues is likely a result of the amount of tissue RNA that was tested, as the result was independent of antibiotic treatment. This method, however, proved to identify *B*. *burgdorferi* specific transcripts from the *in vivo* heart culture, so is likely encumbered by the low numbers of spirochetes in infected monkeys, irrespective of antibiotic treatment.

The lack of success in culturing *B*. *burgdorferi*, also independent of treatment, was not surprising, based on prior studies. Passage through a SCID mouse host did not change this outcome. While the molecular results seemed to indicate dissemination of *B*. *burgdorferi* obtained from XT in SCID mice, we are hesitant to consider this evidence of infectivity, owing to the inconsistency of results. What is perhaps more valuable is that no differences were observed between treated and untreated monkeys in terms of identifying infectious *B*. *burgdorferi*. Spirochetes taken up by xenodiagnostic ticks may require adaptation (>10–14 days) in the tick to become infectious. Studies also indicate that *ospC* expression is associated with migration to the salivary gland and is necessary for primary infection [52]. The known mechanism whereby OspC facilitates primary infection through fostering immune evasion [53] should not be relevant when inoculating the spirochetes into SCID mice, yet the inoculation of *ospC*(-) mutants into SCID mice has been unsuccessful [54].Given that our wild type *B*. *burgdorferi* acquired by xenodiagnostic ticks retain the ability to express *ospC* (also shown by RT-PCR in Fig 4), we cannot rule out the possibility that a tick midgut-adapted phenotype, in the absence of salivation and feeding, contributed to the failure of the *B*. *burgdorferi* acquired from XT to infect SCID mice. In a prior study, when larvae were fed upon infected/treated and infected/untreated mice, the ticks were allowed to molt and were used to feed upon naïve SCID mice. Productive infection was observed with the spirochetes derived from untreated mice but the presence of *B*. *burgdorferi* from antibiotic-treated donor mice in the recipient SCID mice was evident by PCR only [19]. We have not attempted to feed larvae on monkeys because their preferred hosts in nature are small mammals or birds and recovery of contents for analyses is low. However, larvae were utilized in human xenodiagnoses [55], so this may be a viable option for future studies. Nonhuman and human primates are generally considered “dead end” hosts, so adaptation in this host may engender a phenotype of reduced infectivity for subsequent hosts; this is likely an uncontrollable characteristic of the model system, rather than an indication of the viability of the spirochetes.

Our results indicate a lack of uniform predictive serological signatures that could be used to discern between treatment groups and stages of infection. The absence of correlation between serological responses and active infection was apparent from this study, which utilized five diagnostic antigens. Comparison of the acute infection response to the chronic (12 month) response showed that antibody levels to specific antigens waned in some animals and not in others. In a previous study, we characterized the antigen-specific responses over time [48]. In that study, responses to OspC declined as infection proceeded, responses to DbpA remained elevated, and this trend was unaffected by antibiotic treatment (compared to C6). In addition, we previously observed anti-OspA responses with the use of needle inoculation of cultured *B*. *burgdorferi* [35, 48]. In light of a study indicating that PTLDS patients possessed anti-OspA antibodies, we felt it necessary to evaluate this [56]. In the current study, the antibody responses may be more authentic by virtue of the use of tick inoculation. Importantly, we show an absence of anti-OspA responses and that antibody levels to distinct antigens can drop significantly in untreated animals (as for IP67 and IL75) and remain elevated in treated animals (as for IK66 and IH22). This finding illustrates the need for multi-antigen serologic tests.

We place significant value upon the use of xenodiagnosis for detecting persistent *B*. *burgdorferi* because the spirochetes likely need to be viable in order to migrate from the animal tissues to the site of tick feeding. Some investigators have postulated that remnants of the spirochetes [17, 57] could be present following treatment. If these are within blood or skin, perhaps within macrophages [58], they could be taken up during the bloodmeal, but this has not been directly tested and is unlikely to be the case for greater than nine months post-treatment. In addition, the ability to culture *B*. *burgdorferi* from infected primates wanes significantly with duration of infection, irrespective of antibiotic treatment [35, 44]. Following a long period of host adaptation, the spirochetes may not be able to efficiently utilize the nutrients provided by standard BSK culture media. However, the tick provides a natural environment for *B*. *burgdorferi* such that intact spirochetes may be acquired and observed. While it remains possible that the spirochetes detected in xenodiagnostic ticks could be unrelated to the infection, we view this as unlikely. First, we used a highly-specific (OspA) monoclonal antibody for detection, which should only target *B*. *burgdorferi*. Second, our ticks are wild caught at the adult stage but lab-reared from egg mass to nymphs, so with the absence of transovarial transmission, any occurrence (very rare in Louisiana) in adults would not be passed on to progeny. Finally, we fed a portion of our nymphal ticks on clean mice as controls to validate the absence of spirochetes in our tick colony.

From the XT, transcription of 2 genes was also demonstrated, indicating that the spirochetes are viable. These results were clearly found in ticks from 4 of 5 untreated animals and 3 of 5 treated animals. After the second round of xenodiagnosis, the remaining XT contents were instead injected into SCID mice, yet the infectivity of the spirochetes was unclear and independent of antibiotic exposure. We identified, by DFA, few *B*. *burgdorferi* in XT from treated animals after the first round of xenodiagnosis (~7 months p.i.), which may indicate an effect of the antibiotic consistent with reducing the population. However, after the second round of xenodiagnosis (~12 months p.i.), the spirochetes found in XT fed upon treated animals were more frequent and were identified within *in vivo* heart tissue culture and in necropsy sections at a burden indistinguishable from untreated animals. We interpret this as an indication that the doxycycline may reduce the spirochetes to a small, dormant population, but does not rid the primates of viable *B*. *burgdorferi*.

Any one technique applied to the complex dilemma of determining treatment efficacy for a pathogen that is difficult to locate and propagate from its intermediate host may not be sufficient to provide a clear result. We therefore employed multiple tactics to evaluate both the presence and the state of *B*. *burgdorferi* spirochetes that persist after antibiotic treatment of a disseminated infection. Not only did we learn that the spirochetes which persist can be metabolically active (i.e. transcribe RNA), but also that their infectivity is likely reduced in the primate host, which is independent of antibiotic treatment.

As this animal model demonstrates, Lyme can be an insidious disease, with low numbers of spirochetes spread throughout the body. When the animals were examined over a year past initial infection, detection of the pathogen was infrequent, whether or not antibiotic treatment was given. Were the spirochetes actively dividing and spreading during infection, the disease would be more obvious and possibly more easily diagnosed. Bacterial pathogens are well known to cause tissue damage by colonization, induction of intense inflammation, invasion of host cells, and production of toxins [59]. Borrelia, rather, induces milder inflammatory responses and has mastered evasion of host immunity so as to cause moderate disease without being recognized and eliminated. The use of nonhuman primates to model this disease provides the most accurate representation of human Lyme disease as demonstrated in this work. Here we observed not only variation in the objective clinical manifestations, such as development of the EM rash, but also derived evidence for pauci-spirochetal colonization and persistence that was unaffected by doxycycline treatment. We show that primates infected with the same genotypic strain of *B*. *burgdorferi* generate widely variable antibody responses that change significantly over time despite persistent infection. The variation typified by this animal model should be exploited to uncover host differences and biomarkers indicative of responses to infection and treatment.

## Supporting information

S1 Fig Immunoblot using serum from animal IK14 to detect reactivity to *B*. *burgdorferi* antigens. Week 16 post-inoculation serum was tested for IgM and IgG antibodies; pre-immune serum was tested for IgG. Proteins that were separated and fixed to the membrane included *B*. *burgdorferi* lysate (L) and recombinant proteins OspC, OspA, OppA-2 and DbpA (rP). M = molecular weight marker.
(TIF)

S2 Fig Anti-OspA antibody levels in monkeys over the course of infection. Each graph represents one animal, with the untreated monkeys shown in the left column and the treated animals on the right. Animals were treated with doxycycline between weeks 16–20. Vertical axis: MFI = mean florescent intensity. Shown is the mean ±SEM for each time point.
(TIF)

S3 Fig Inflammation in skin following second tick feeding on macaques. Erythematous papules at the sites of tick feeding are shown in Panel A. Skin biopsies were taken distal to the bite site (Panel B) and within the papule (Panel C). The tick feeding site shows ulceration at the location of hypostome penetration with mixed neutrophilic/eosinophilic/ histiocytic inflammation and necrosis extending into the deep dermis accompanied by disorganization of the collagen bundles (Panel D). Mild edema and hemorrhage are also present immediately adjacent to the bite site.
(TIF)

S4 Fig RT-PCR of tissues from SCID mice inoculated with contents of XT derived from macaques. Set 1 is from mice inoculated with midgut contents of ticks fed upon monkeys (xeno/scid). Set 2 is tissues from mice inoculated with midgut contents of ticks fed upon monkeys and those mice were then also subjected to xenodiagnosis (xeno/scid/xeno). Clear positives are marked with (*) and potential positive results are marked with (^). Controls including no template control (NTC), uninfected monkey tissue (-Mk) and uninfected SCID mice (Neg scid) are included.
(TIF)

S1 Table Serum doxycycline concentrations obtained during the treatment period. (DOCX)

S2 Table Blood chemistry, CBC and physical exam results. (DOCX)
