# Supplementary figures and images for: Variable manifestations, diverse seroreactivity and post-treatment persistence in non-human primates exposed to Borrelia burgdorferi by tick feeding
Source: PLoS One. 2017 Dec 13;12(12):e0189071. doi: 10.1371/journal.pone.0189071 (PMC5728523; doi:10.1371/journal.pone.0189071)

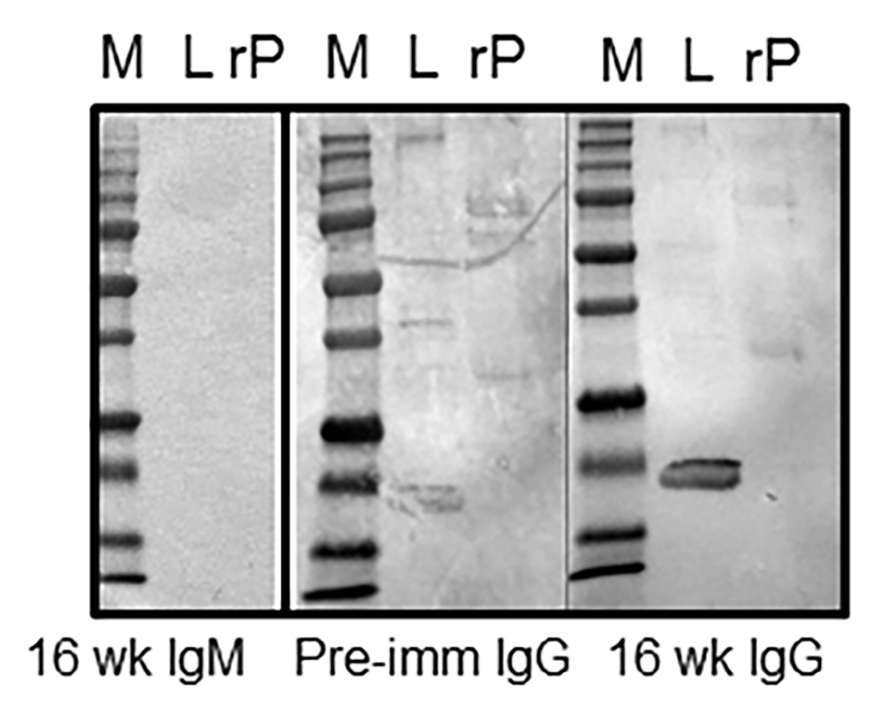

Supplement: S1 Fig — Week 16 post-inoculation serum was tested for IgM and IgG antibodies; pre-immune serum was tested for IgG. Proteins that were separated and fixed to the membrane included B. burgdorferi lysate (L) and recombinant proteins OspC, OspA, OppA-2 and DbpA (rP). M = molecular weight marker. (TIF) [file pone.0189071.s001.tif]

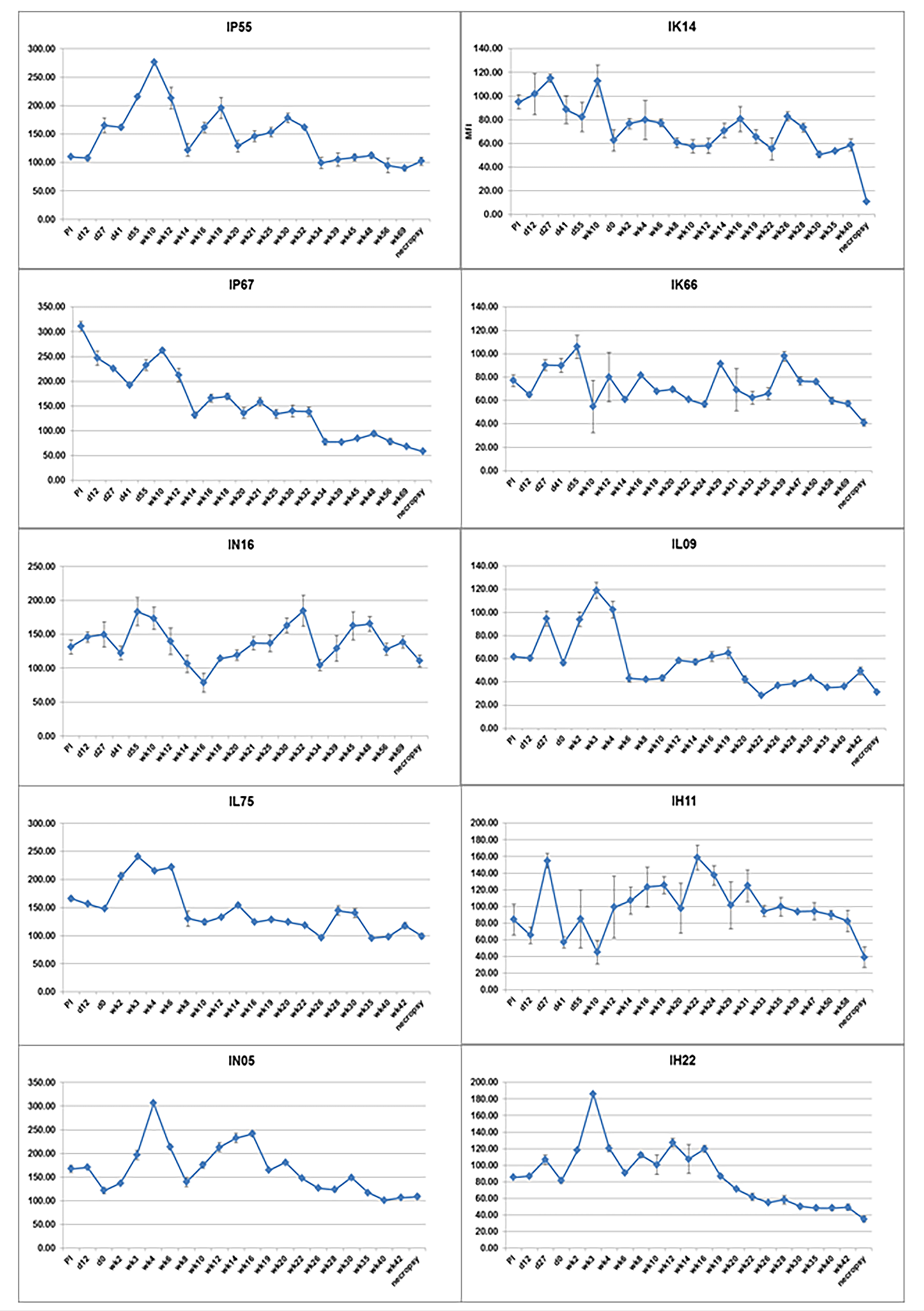

Supplement: S2 Fig — Each graph represents one animal, with the untreated monkeys shown in the left column and the treated animals on the right. Animals were treated with doxycycline between weeks 16–20. Vertical axis: MFI = mean florescent intensity. Shown is the mean ±SEM for each time point. (TIF) [file pone.0189071.s002.tif]

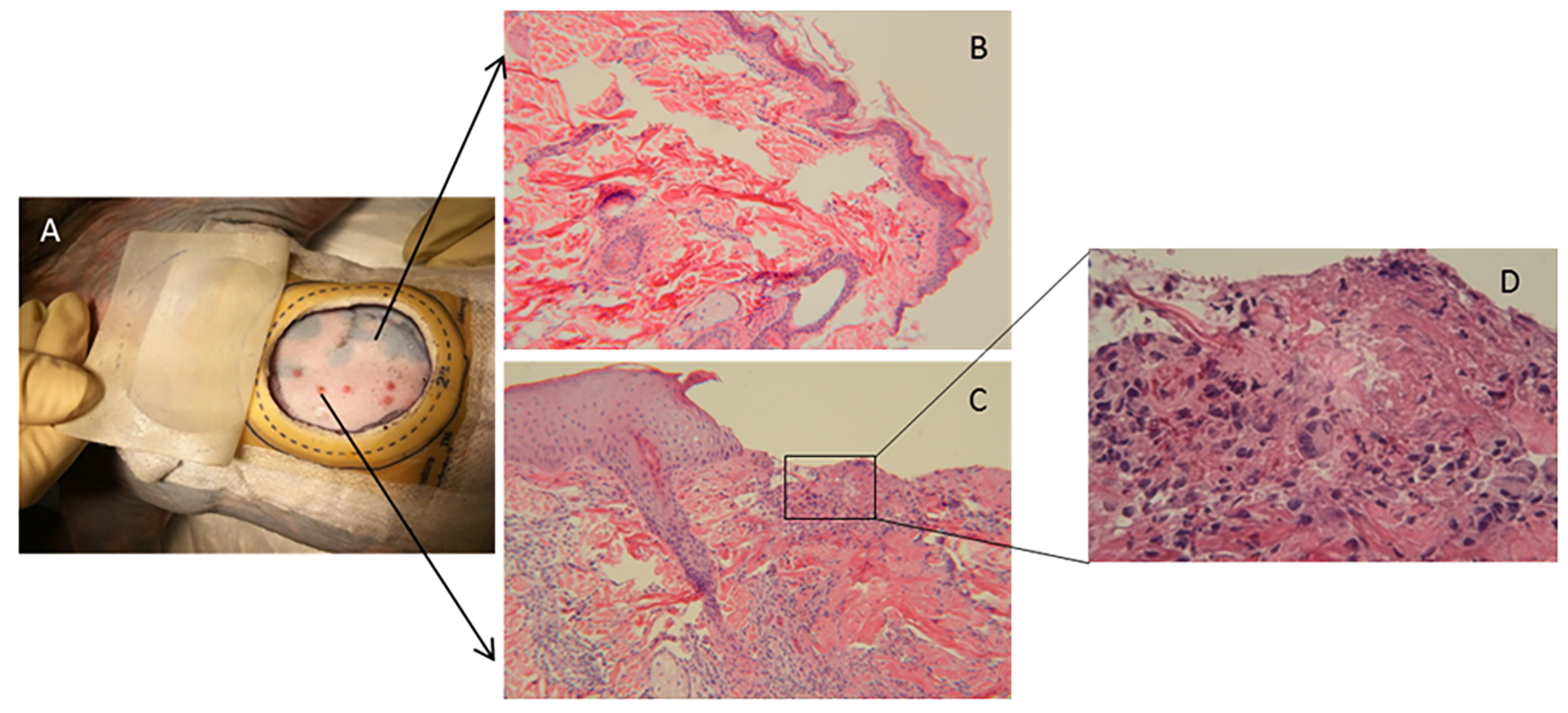

Supplement: S3 Fig — Erythematous papules at the sites of tick feeding are shown in Panel A. Skin biopsies were taken distal to the bite site (Panel B) and within the papule (Panel C). The tick feeding site shows ulceration at the location of hypostome penetration with mixed neutrophilic/eosinophilic/ histiocytic inflammation and necrosis extending into the deep dermis accompanied by disorganization of the collagen bundles (Panel D). Mild edema and hemorrhage are also present immediately adjacent to the bite site. (TIF) [file pone.0189071.s003.tif]

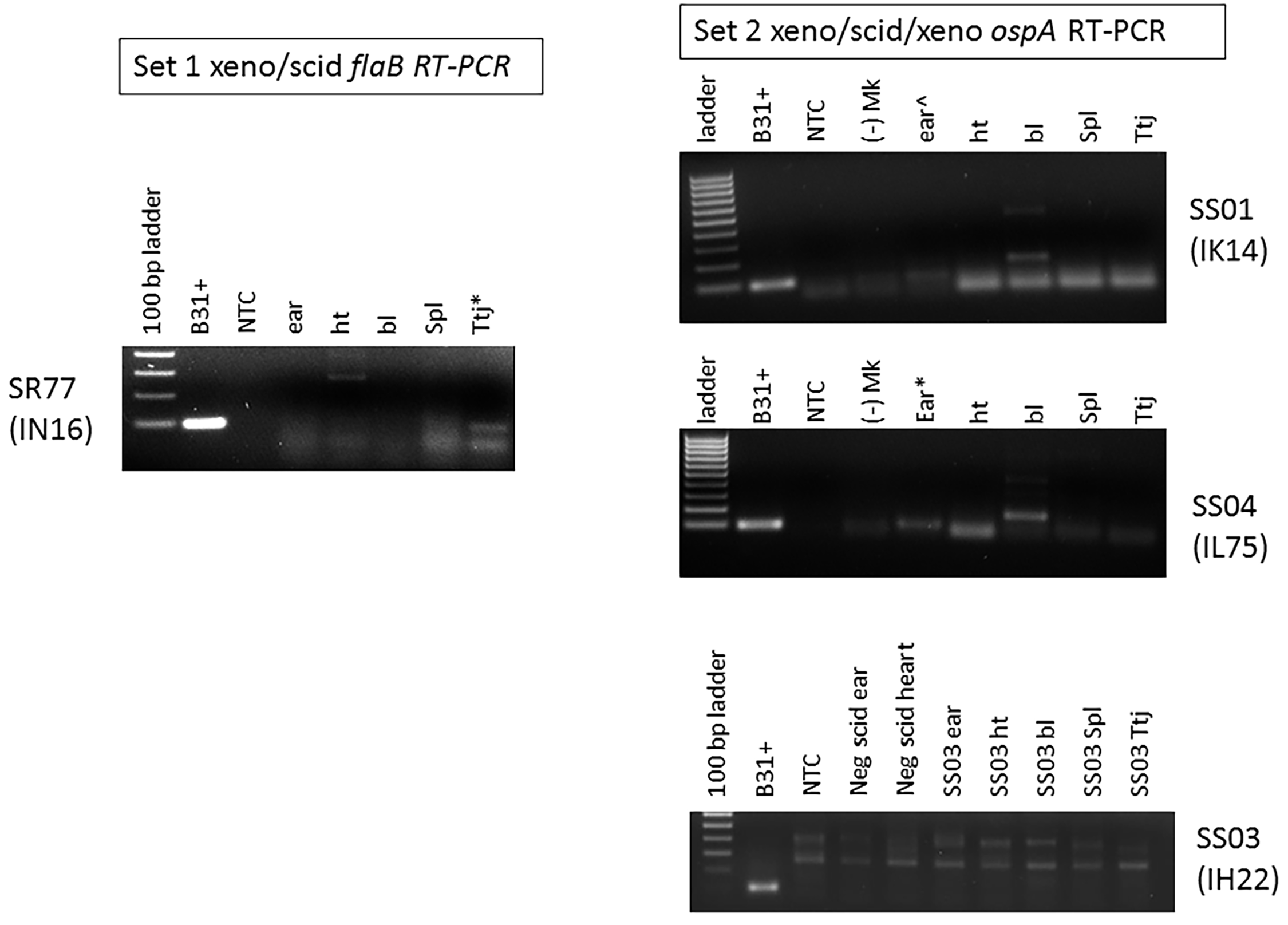

Supplement: S4 Fig — Set 1 is from mice inoculated with midgut contents of ticks fed upon monkeys (xeno/scid). Set 2 is tissues from mice inoculated with midgut contents of ticks fed upon monkeys and those mice were then also subjected to xenodiagnosis (xeno/scid/xeno). Clear positives are marked with (*) and potential positive results are marked with (^). Controls including no template control (NTC), uninfected monkey tissue (-Mk) and uninfected SCID mice (Neg scid) are included. (TIF) [file pone.0189071.s004.tif]
