# Supplementary material for: Variable manifestations, diverse seroreactivity and post-treatment persistence in non-human primates exposed to Borrelia burgdorferi by tick feeding
Source: PLoS One. 2017 Dec 13;12(12):e0189071. doi: 10.1371/journal.pone.0189071 (PMC5728523; doi:10.1371/journal.pone.0189071)
Supplement: S1 Table — (DOCX) [file pone.0189071.s005.docx]

| **Monkey** | **Time point** | **Extrapolated Serum Doxycycline Concentration (µg/mL)*** |
| --- | --- | --- |
| IK14 | Week 19  Week 20 | 0.1 ± 0  0.255 ± 0.219 |
| IL09 | Week 19  Week 20 | 1.38 ± 1.131  0.34 ± 0.057 |
| IH22 | Week 19  Week 20 | 1.865 ± 1.25  1.795 ± 2.397 |
| IH11 | Week 18  Week 20 | 1.081 ± 0.508  0.998 ± 0.866 |
| IK66 | Week 18  Week 20 | 1.081 ± 0.508  0.984 ± 0.886 |
| IL75 (untreated) | Week 18 | 0 µg/mL |
|  |  |  |

**S1 Table. Serum doxycycline concentrations obtained during the treatment period.**

*Mean and SD of two independent assays
