# Supplementary material for: Variable manifestations, diverse seroreactivity and post-treatment persistence in non-human primates exposed to Borrelia burgdorferi by tick feeding
Source: PLoS One. 2017 Dec 13;12(12):e0189071. doi: 10.1371/journal.pone.0189071 (PMC5728523; doi:10.1371/journal.pone.0189071)
Supplement: S2 Table — (DOCX) [file pone.0189071.s006.docx]

**S2 Table . Blood Chemistry, CBC and Physical Exam Results**

|  | **Liver enzymes**  (pre-inf./2 months post) | | **Blood counts**  (pre-inf./2 months post) | | | **Exam notes** | **Observations/Pathology report** |
| --- | --- | --- | --- | --- | --- | --- | --- |
| **Animal #** | **ALT** | **AST** | **%Neu** | **%Lym** | **%Mon** |  |  |
| **IK14 (treated)** | 32/32 | 38/47 | 42.5/21.7 | 52.6/67.0 | 2.0/2.2 | Erythema migrans (observed 2 weeks after tick removal); mild submandibular lymphadenopathy (22 weeks p.i.) | Mesenteric lymph node hyperplasia |
| **IL09 (treated)** | 27/25 | 31/33 | 46.6/45.9 | 43.5/43.8 | 6.4/5.0 | Enlarged inguinal and axillary LN (28 weeks p.i.); axillary LN (8 weeks p.i.) | Mes/Axillary lymph node hyperplasia  Lung Inflammation  Knee synovium-pericapsular stromal inflammation |
| **IH22 (treated)** | 22/22 | 28/37 | 36.8/32.0 | 58.2/62.4 | 2.8/3.1 | Mild axillary and peripheral lymphadenopathy (1-2 weeks p.i.); abdominal seborrhea—multiple 2-5 mm patches of slightly raised, erythematous areas (2 weeks p.i.); mild submandibular lymphadenopathy (22 weeks p.i.) | Spleen hyperplasia  Lung inflammation |
| **IL75 (untreated)** | 35/34 | 37/37 | 44.6/45.7 | 48.1/48.4 | 3.4/3.8 | Moderate erythematous rash on caudal abdomen and inguinal region (2 weeks p.i.; 2 months p.i.) axillary and inguinal LN mildly enlarged (2 months p.i.) | Sural PN-perineural lymphoid aggregate  Lung-inflammatory foci, hyperplasia  LN hyperplasia  Spleen hyperplasia |
| **IN05 (untreated)** | 32/33 | 35/37 | 51.7/51.6 | 44.5/44.4 | 2.5/3.1 | 10 papules at sites of tick feeding; axillary lymphadenopathy 1 week p.i.), peripheral lymphadenopathy 2 weeks p.i., mild splenomegaly 16 weeks p.i. | Brain-inflammatory lesions  Ganglia-Ca2+ deposits (nerve cell damage)  Skeletal muscle (arm)-focal granuloma  Lung hyperplasia |
|  | **Liver enzymes**  (pre-inf./4 months post) | | **Blood counts**  (pre-inf./4 months post) | | |  |  |
| **IH11 (treated)** | 16/18 | 32/40 | 45.1/20.3 | 47.7/73.0 | 4.2/4.1 | One tick bite lesion (not EM):axillary lymphadenopathy (2 weeks) | Cervical nerve root-focal inflammatory lesion  Lung-hyperplasia  Heart-focal Inflammatory lesion |
| **IK66 (treated)** | 26/28 | 31/33 | 43.7/48.2 | 47.3/43.6 | 4.1/5.2 | Three tick bite lesions (not EM): left axillary lymphadenopathy (2 weeks; 6 months p.i.) | Skeletal muscle (thigh)-focal inflammatory lesion |
| **IP67 (untreated)** | 25/41 | 25/48 | 55.8/70.9 | 36.7/25.1 | 2.9/3.1 | 3 pinpoint lesions where ticks fed (1 week p.i.), mild petechial lesions caudal dorsum; (7 months p.i.) | None observed |
| **IN16 (untreated)** | 24/37 | 28/44 | 61.7/51.8 | 26.9/39.8 | 3.6/4.7 | axillary lymphomegaly (4 months) | LN hyperplasia  Lung hyperplasia |
| **IP55 (untreated)** | 36/34 | 40/41 | 72.3/74.4 | 23.0/20.5 | 2.6/3.3 | axillary lymphadenopathy (1 week) | PN-perineural inflammation  Reduced activity/weak/depressed; mild rapid breathing; animal had tarsal wound/infection (6 months p.i.); abdominal dermatitis (7 months p.i.) |
